# Body Dysmorphic Disorder or Psychosis: How to Draw the Line? A Case Report

**DOI:** 10.1192/j.eurpsy.2026.12123

**Published:** 2026-07-31

**Authors:** Y. Yaich, C. Bey, B. Abassi, G. Amri, A. Hakiri, R. Ghachem

**Affiliations:** Department of Psychiatry B, University Hospital Razi, Manouba, Tunisia

## Abstract

**Introduction:**

Body dysmorphic disorder (BDD) is characterized by intrusive appearance-related concerns and is now classified within the obsessive–compulsive spectrum. A substantial proportion of patients, however, hold their beliefs with delusional conviction, blurring the boundary with psychotic disorders.

**Objectives:**

This report highlights the diagnostic challenges in distinguishing body dysmorphic disorder with delusional beliefs from primary psychotic disorders through a detailed clinical observation of a patient treated in the psychiatry department at Razi Hospital.

**Methods:**

Mr. S.N, a 35-year-old male, was hospitalized in June 2025 due to suicidal ideation. He had no family history of mental illness. His personal history revealed multiple consultations with psychiatrists, during which he was prescribed antidepressants, though he discontinued them after a short period. The onset of his symptoms began in 2013, marked by increased irritability, social withdrawal, and a growing obsession with his appearance. By 2021, Mr S.N expressed concerns about his physical condition, believing that others were fixated on his “crooked” smile. He engaged in excessive mirror-checking, spending up to 15 minutes multiple times a day scrutinizing his reflection after each social interaction. His physical examination revealed no abnormalities. Initially, a diagnosis of body dysmorphic disorder was considered due to his somatic delusion about his perceived crooked smile. However, over time his presentation became increasingly atypical: he described being scrutinized from a distance, repeatedly monitored by others, and engaged in physical altercations. He showed marked ambivalence, at times acknowledging his thoughts as illness-related, then immediately expressing full conviction. His behavior was strikingly odd, including cutting holes into self-report questionnaires, pacing anxiously in waiting rooms, and making repeated phone calls to request medication changes. He also reported suicidal fears, stating that if left alone he might wander to a mountain or train tracks to harm himself, while simultaneously demanding hospitalization for protection but insisting on immediate discharge once admitted. These features, extending beyond a circumscribed preoccupation with appearance and coupled with disorganized help-seeking and suicidal threats, led us to consider a psychotic disorder.

**Results:**

The main difference between BDD and psychosis lies in delusional content and expression: while BDD focuses on appearance with reassurance or neutralizing behaviors, psychosis involves bizarre, often appearance-related delusions, expressed in unusual ways. Psychotic patients also exhibit extreme anxiety, ambivalence, and odd or disorganized behaviors reflecting the unusual elaboration of their beliefs.

**Conclusions:**

Careful evaluation of delusional content, behavioral oddities, and distress is essential to differentiate BDD from psychosis and guide appropriate treatment.

**Disclosure of Interest:**

None Declared

